# Preparation, Biodistribution and Neurotoxicity of Liposomal Cisplatin following Convection Enhanced Delivery in Normal and F98 Glioma Bearing Rats

**DOI:** 10.1371/journal.pone.0048752

**Published:** 2012-11-13

**Authors:** Tianyao Huo, Rolf F. Barth, Weilian Yang, Robin J. Nakkula, Rumiana Koynova, Boris Tenchov, Abhik Ray Chaudhury, Lawrence Agius, Teni Boulikas, Helene Elleaume, Robert J. Lee

**Affiliations:** 1 Department of Pathology, The Ohio State University, Columbus, Ohio, United States of America; 2 Division of Pharmaceutics, College of Pharmacy, The Ohio State University, Columbus, Ohio, United States of America; 3 Department of Pathology, Mater Dei Hospital and University of Malta Medical School, Msida, Malta; 4 Regulon Inc., Mountain View, California, United States of America; 5 European Synchrotron Radiation Facility, Grenoble, France; Wake Forest University School of Medicine, United States of America

## Abstract

The purpose of this study was to evaluate two novel liposomal formulations of cisplatin as potential therapeutic agents for treatment of the F98 rat glioma. The first was a commercially produced agent, Lipoplatin™, which currently is in a Phase III clinical trial for treatment of non-small cell lung cancer (NSCLC). The second, produced in our laboratory, was based on the ability of cisplatin to form coordination complexes with lipid cholesteryl hemisuccinate (CHEMS). The *in vitro* tumoricidal activity of the former previously has been described in detail by other investigators. The CHEMS liposomal formulation had a Pt loading efficiency of 25% and showed more potent *in vitro* cytotoxicity against F98 glioma cells than free cisplatin at 24 h. *In vivo* CHEMS liposomes showed high retention at 24 h after intracerebral (i.c.) convection enhanced delivery (CED) to F98 glioma bearing rats. Neurotoxicologic studies were carried out in non-tumor bearing Fischer rats following i.c. CED of Lipoplatin™ or CHEMS liposomes or their “hollow” counterparts. Unexpectedly, Lipoplatin™ was highly neurotoxic when given i.c. by CED and resulted in death immediately following or within a few days after administration. Similarly “hollow” Lipoplatin™ liposomes showed similar neurotoxicity indicating that this was due to the liposomes themselves rather than the cisplatin. This was particularly surprising since Lipoplatin™ has been well tolerated when administered intravenously. In contrast, CHEMS liposomes and their “hollow” counterparts were clinically well tolerated. However, a variety of dose dependent neuropathologic changes from none to severe were seen at either 10 or 14 d following their administration. These findings suggest that further refinements in the design and formulation of cisplatin containing liposomes will be required before they can be administered i.c. by CED for the treatment of brain tumors and that a formulation that may be safe when given systemically may be highly neurotoxic when administered directly into the brain.

## Introduction

Platinum containing drugs are widely used chemotherapeutic agents for the treatment of a variety of human cancers [1], [2]. However, their renal, gastro-intestinal and neurotoxicity, rapid binding to plasma proteins, and poor penetration of the central nervous system (CNS) have limited their use for the treatment of brain tumors [2]–[4]. We recently have reported that intracerebral (i.c.) convection enhanced delivery (CED) or osmotic pump infusion of carboplatin in combination with radiotherapy (RT), produced a 2.5 to 3.6 fold increase in the mean survival time (MST) of F98 glioma bearing rats with a subset of cured animals [5]. However, the wide range of survival times (37–180 days) suggested that there was non-homogenous distribution of the drug within the tumors. This occurred despite the fact that i.c. CED of 20 µg of carboplatin resulted in a tumor drug concentration (10.4 µg/g) equivalent to that observed following i.v. administration of a 1000× greater dose (20 mg or 20,000 µg) to F98 glioma bearing rats. These observations suggested to us that a liposomal formulation of the drug might result in not only more sustained release, but also improved microdistribution within the tumor [6], [7].

Liposomes have been used clinically to deliver a variety of anticancer drugs including cisplatin [8]–[11], but not for the treatment of brain tumors. Bankiewicz [12]–[14] and Dickinson [15], [16] and their research teams have carried out extensive studies in rodents, dogs and primates to evaluate CED of liposomes, loaded with either tracers or therapeutic agents for the treatment of brain tumors. Loading cisplatin into liposomes potentially could reduce its systemic toxicity and improve its microdistribution within brain tumors following CED [17]. For the design and preparation of liposomal nanovehicles, it is important to achieve a balance between their stability, encapsulation efficiency, drug release, i.c. drug distribution, and tumoricidal activity. This is especially true if they are to be used for the treatment of brain tumors, where the blood-brain barrier (BBB) severely limits tumor uptake following systemic administration [18]. This could be circumvented by directly administering them i.c. by means of CED, if the delivery parameters can be optimized [19]. Readers interested in more detailed information relating to CED of liposomes for the treatment of brain tumors are referred to a recent review on this topic [20].

In the present study we initially evaluated a proprietary liposomal formulation of cisplatin (Lipoplatin™, Regulon Inc. Mountain View, CA) [6]–[11] and their “hollow” counterparts in non-tumor bearing Fischer rats. These unexpectedly proved to be highly neurotoxic, and served as an impetus for us to develop a formulation that would be better tolerated following i.c. CED. In this report we describe a novel liposomal cisplatin formulation that we have prepared, which was based on the ability of cisplatin to form coordination complexes with lipid cholesteryl hemisuccinate (CHEMS) [21]. These liposomes showed increased *in vitro* cytotoxicity against F98 glioma cells compared to free cisplatin and tumor retention in F98 glioma bearing rats. However, neurotoxicologic studies, carried out in non-tumor bearing animals, showed that the CHEMS liposomes and their “hollow” counterparts produced a variety of neuropathologic changes ranging from minimal to severe. These findings indicated that they were not suitable for therapy studies and that further work will be required to produce liposomal cisplatin containing nanovehicles that will be well tolerated when administered i.c. by CED to F98 glioma bearing rats.

## Materials and Methods

### Chemicals

Egg yolk phosphatidylcholine (egg PC), and 1,2-distearoyl-*sn*-glycero-3-phosphoethanolamine-N- [methoxy (polyethylene glycol)-2000] ammonium salt (mPEG-DSPE) were purchased from Avanti Polar Lipids (Alabaster, AL). Cholesteryl hemisuccinate (CHEMS, MW 486 Da) and cisplatin (MW 300.04 Da) were purchased from Fisher Scientific, Pittsburgh, PA. An aqueous solution of cisplatin (5 mM) was prepared by incubating it overnight in the dark at 37°C in order to reach full equilibration.

### Preparation and Characterization of Lipoplatin™ and CHEMS Liposomes

Lipoplatin™ is a liposomal formulation of cisplatin, composed of the lipids 1, 2-dipalmitoyl-sn-glycero-3-[phospho-rac-(1-glycerol)] (sodium salt), also known as dipalmitoyl phosphatidyl glycerol (DPPG, MW 745Da), soy phosphatidyl choline (SPC-3, MW 790 Da), cholesterol (MW, 386.66 Da) and mPEG-DSPE. It was produced in two steps: *First* liposomes composed of SPC-3, cholesterol and mPEG-DSPE were prepared. *Second*, reverse micelles between the cisplatin dihydroxy form (positively charged) and DPPG (negatively charged) were formed in an ethanolic solution, which then was engulfed by the liposomes from step 1 by mixing them under proprietary conditions. The resulting preparation was a sterile, non-pyrogenic opaque liquid intended for intravenous injection [8]. The “hollow” Lipoplatin liposomes were produced in a similar manner but did not contain any cisplatin.

**Figure 1:**
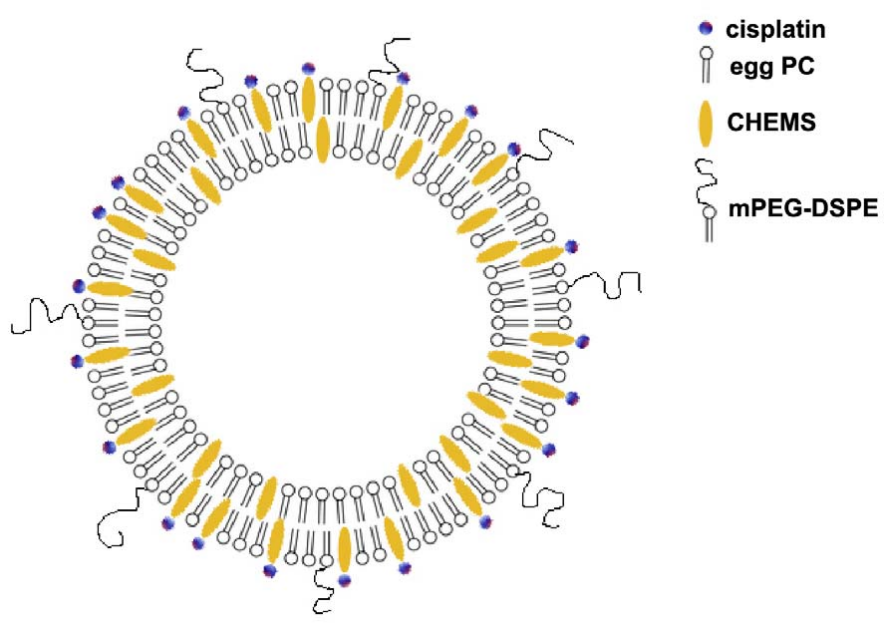
Schematic representation of a liposome containing CHEMS-cisplatin complexes prepared by the addition of a cisplatin solution to preformed egg PC/CHEMS/mPEG-DSPE liposomes.

To prepare CHEMS liposomes egg PC, CHEMS and mPEG-DSPE (20∶10∶1, w/w) were mixed in chloroform and dried into a thin film by evaporating the bulk of the solvent under nitrogen, and the residual solvent was removed under vacuum. A 10% by weight (wt%) aqueous sucrose solution (pH 8.0), was added and the lipid film was hydrated overnight at ambient temperature and finally for 1 h at 37°C. The lipid dispersion was sonicated for 1 min in a bath sonicator to generate liposomes, and then processed by a high-pressure EmulsiFlex-C5 homogenizer (Avestin, Inc., Ottawa, Canada) for 10 min, in order to reduce particle size. Cisplatin (5 mM in 10 wt% sucrose, pH 8.0) was added to the liposomes (CHEMS: cisplatin ∼1∶2.5 mol/mol) and it was incubated at 37°C for 24 h. The unbound cisplatin was removed by hollow-fiber diafiltration (MicroKros Spectrum Laboratories, Inc., Rancho Dominguez, CA), sterilized by passing them through a 0.45 µm filter (Millipore Corp., Billerica, MA) and then freeze-dried. A schematic diagram of the resulting purified CHEMS liposomes are shown in Fig. 1. The “hollow” CHEMS liposomes were prepared in a similar way but did not contain any cisplatin.

### Determination of Size, Zeta Potential and *in vitro* Tumoricidal Activity

The size distribution of the Lipoplatin™ was determined by dynamic light scattering on Beckman Coulter N4+ particle size analyzer and for CHEMS liposomes on a NICOMP Submicron Particle Sizer. All particle size data refer to volume-weighted distributions. The zeta potentials (ζ) of the liposomes were determined on a ZetaPALS instrument (Brookhaven Instruments Corp., Holtsville, NY). CHEMS liposomes, at a dose of 15 µg cisplatin per flask, were added to F98 rat glioma cells (CRL-2397, ATCC), propagated in T-75 flasks. Cells were incubated at 37°C for 1, 2, and 4 h. they then were trypsinized and harvested, followed by centrifugation to obtain cell pellets. These were resuspended in 2 ml of phosphate buffered saline (PBS, pH 7.4) and then processed for Pt determinations by means of Inductively Coupled Plasma-Optical Emission Spectroscopy (ICP-OES) at a later date [5]. To assess the *in vitro* cytotoxicity of free cisplatin, Lipoplatin™ and CHEMS liposomes and their “hollow” counterparts, F98 glioma cells were treated with varying concentrations of either for 4 or 24 hrs. The cells then were plated out in petri dishes, 3 replicates for each concentration and at 7 days the cultures were terminated and colonies were enumerated as previously described [5]. Colonies with >50 cells were enumerated under a dissecting microscope and the surviving fractions (S.F.) were calculated.

### Neurotoxicologic Studies

All of the animal studies were carried out in strict accordance with the recommendations in the *Guide for the Care and Use of Laboratory Animals of the National Institutes of Health* and our protocol was approved by the Institutional Animal Care and Use Committee of The Ohio State University (Permit #: A-3261-01 and IACUC protocol number 2007A0261-R1).

Neurotoxicologic studies were carried out in non-tumor bearing male Fischer rats. Initially, a dose escalation study was performed using either Lipoplatin™ or CHEMS liposomes. The former contained 0.45, 0.90, 1.80 or 2.70 µg of cisplatin, respectively, and the latter contained 0.48 µg/µL of cisplatin in 10, 15 and 20 µL. Non-cisplatin containing (“hollow”) liposomes of each type were evaluated as vehicle controls. All of these agents were administered into the striatum of the right cerebral hemisphere by CED over 30 min [5] using a 28 gauge needle as a cannula with 3 animals for each concentration. Those rats that received Lipoplatin™ and did not die acutely were monitored daily and euthanized at 4 d following CED. Animals that received CHEMS liposomes were monitored clinically and weighed 3X per week until they were euthanized at either 10 or 14 d following administration. The brains of all animals were removed and processed for neuropathologic examination [5].

### Biodistribution Studies in F98 Glioma Bearing Rats

The F98 rat glioma was derived from an undifferentiated neoplasm that was induced in the progeny of a pregnant CD Fischer rat that had received an injection of N-ethyl-N-nitrosourea. It has been propagated *in vitro* and *in vivo* since 1971 and, as described in a recent review [22], it has been used in a wide variety of studies in experimental neuro-oncology. F98 cells were grown in Dulbecco’s modified Eagle’s medium (DMEM) (Gibco, Grand Island, NY) supplemented with 10% fetal bovine serum (FBS) (Hyclone, Logan, UT), 100 units/mL penicillin, 100 µg/mL streptomycin and 2 mM L-glutamine. Fischer rats (Animal Production Branch National Cancer Institute, Frederick, MD) weighing 220–240 g were used in the present study. A stereotactic implantation procedure, which has been described in detail elsewhere [23], was employed. F98 cells at a concentration of 10^5^ cells/10 µl in DMEM containing low gelling temperature agarose were injected stereotactically into the right caudate nucleus over 10–15 s through a small entry port of the plastic screw.

Thirteen days later, at which time the tumors had attained volumes of ∼25–30 mm [5], biodistribution studies were initiated. CHEMS liposomes were diluted in normal saline and administered to 8 rats by CED over 30 min (9.6 µg/10 µl). Immediately following or 24 h after CED, 4 rats were euthanized, their brains were removed, the tumors were carefully dissected out from surrounding normal brain and they were weighed, and stored at −70°C. Similarly, a 1 mm zone surrounding the excised tumor was dissected out, and this was designated as “brain around tumor” (BAT). The remaining right cerebral hemisphere and the left hemisphere were designated “normal” brain. Platinum determinations in all of these tissues were performed at a later date by means of ICP-OES. Based on Pt uptake values, the concentrations of cisplatin (MW 300.05 Da), were calculated by multiplying the Pt values by 1.54. Platinum retention in the F98 glioma after CED of CHEMS liposomes was calculated as % injected dose (ID)/g tumor.

### Statistical Evaluation of Data

For data obtained in the *in vitro* cytotoxicity studies, the means and standard deviations (SD) of surviving fractions of F98 glioma cells were calculated. Data were first log transformed and then fit to a quadratic function. The data also were used to test the difference in S.F. of the cells using a one-way Analysis of Variance (ANOVA), followed by a *post hoc* test using Tukey’s method [24]. Analysis of the differences in *in vitro* toxicity of Lipoplatin™ and CHEMS liposomes, and their “hollow” counterparts was determined by means of a two-sample t-test. For the biodistribution data, the means and SD were computed for cisplatin in tumor, brain around tumor (BAT), ipsilateral (tumor bearing) and contralateral (non-tumor bearing) cerebral hemispheres and blood. A two-sample t-test was used to compare Pt concentrations in these tissues at 0 or 24 hours following CED. For retention of Pt in F98 glioma bearing rats, a two-way ANOVA was used to test for differences between free carboplatin and CHEMS liposomes and those between two time points (0 and 24 h). Differences were considered significant if the *P* value was <0.05.

## Results

### Preparation and Characterization of CHEMS Liposomes

As a result of the addition of cisplatin, the zeta (ζ) potential of the lipid vesicles decreased from −30.9 mV to −7.9 mV, thereby confirming that it had bound to the liposomes (Table 1). Assuming a uniform distribution of CHEMS on the two sides of the lipid bilayer, the molar ratio of the CHEMS in the outer/inner layer should have been close to 0.6 for the observed liposome size. Based on the procedure that we employed, only the outer layer of the liposomal membrane was accessible for cisplatin and a maximum molar ratio of the 1∶0.6 was attained for CHEMS/cisplatin liposomes (Table 1). This could either indicate that virtually all CHEMS molecules in the outer lipid layer had reacted with cisplatin or alternatively that coordination compounds had been formed at 1∶1 stoichiometry.

**Table 1:** Formulations of liposomal cisplatin preparations.

LipidComposition	Cisplatin loading(mol/mol)	Loadingefficiency	Particle size(nm)	Zeta potential(mV)
Egg PC:CHEMS:mPEG-	CHEMS: cisplatin	25%	55.2±34.3^a^	−30.9±0.4^a^
DSPE	1 : 2.5 (initial)		56.7±20.8^b^	−7.9±1.1^b^
	1 : 0.6 (final)		52.3±18.2^c^	−8.3±0.7^c^
Lipoplatin™				−54.7±5^d^
DPPG: SPC-3:				−49.6±5^e^
cholesterol:				
mPEG-DSPE				

a Hollow liposomes (before cisplatin loading).

b Liposome/cisplatin measured immediately after preparation.

c After lyophilization, 10 d storage at ambient temperature, and resuspension.

d In water.

e In 5% dextrose.

### *In vitro* Tumoricidal Activity of Either Free Cisplatin or Cisplatin Containing Liposomes

**Figure 2:**
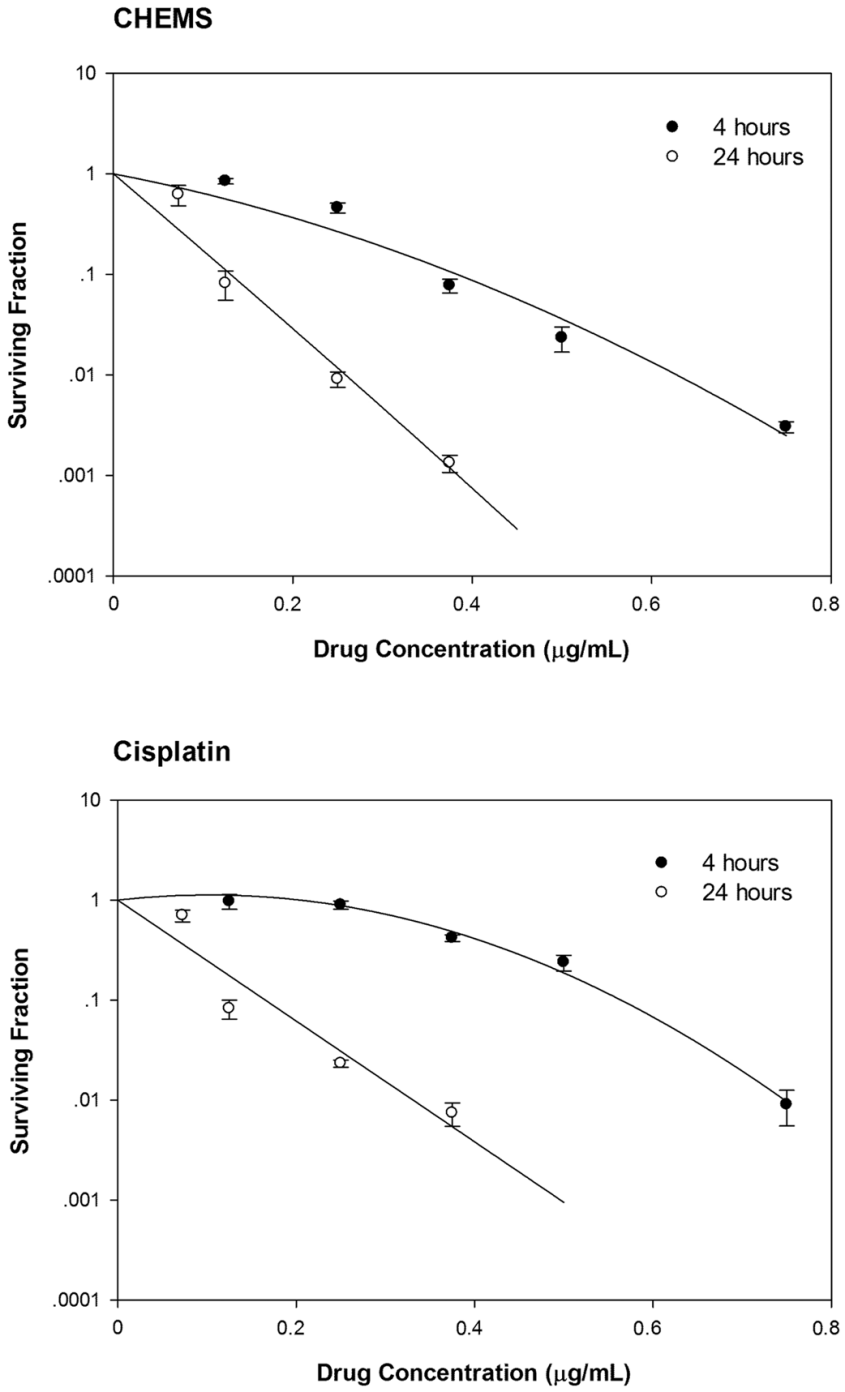
Clonogenic survival of F98 glioma cells following treatment with either free cisplatin or liposomal cisplatin for 4 or 24 h. Surviving fractions (S.Fs) were determined for the F98 glioma cells treated with (A) CHEMS lipsomes, (B) free cisplatin following a 4 h (•) or 24 h (○) exposure. Each data point represents the mean of 3 replicates ± the standard deviation.

The *in vitro* tumoricidal activity of Lipoplatin™ previously has been described in detail elsewhere by Paquette and his research team [25]. Clonogenic survival data for F98 glioma cells that had been exposed to free cisplatin or either Lipoplatin™ or CHEMS liposomes or their “hollow” counterparts at 4 or 24 h are shown in Fig. 2 and Table 2. The IC_90_ of cisplatin and CHEMS at 4 h were 0.600 and 0.383 µg/mL, respectively, *versus* 0.335 and 0.259 µg/mL at 24 h. The S.F. of F98 cells were 0.13% following 24 h treatment with CHEMS liposomes containing 0.375 µg/mL cisplatin, *versus* 0.74% for free cisplatin. Thus, the CHEMS showed more potent cytotoxicity than free cisplatin at 24 h.

**Table 2:** Comparison of the toxicity against F98 glioma cells of Lipoplatin™ and CHEMS cisplatin liposomes and their “hollow” counterparts.

Test agent	Cisplatin conc(µg/ml)	Surviving fraction(mean ± SD)	P-value*
Lipoplatin™	3	0.51±0.13	0.42
“Hollow”Regulon liposomes	0	0.43±0.05	
CHEMS liposomes	0.5	0.023±0.003	0.02
“Hollow” CHEMS liposomes	0	0.59±0.13	

a F98 glioma cells were exposed to the test agent for 4 hours, following which clonogenic assays were carried.

b Surviving fractions were determined following 7 days incubation at 37°C in a CO_2_ incubator.

* P-value is computed using two-sample t-test.

**Figure 3:**
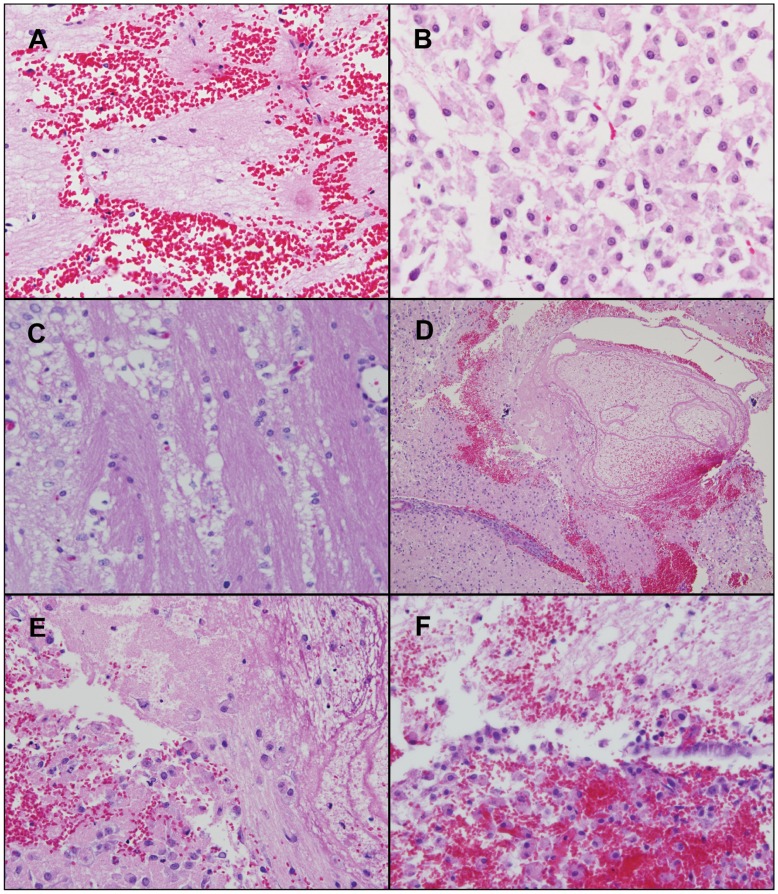
Neuropathologic changes associated with i.c. CED of Lipoplatin™ or its “hollow” counterparts or free cisplatin (A). (A) Lipoplatin™ (1.8 µg). Rat was euthanized 2 hr after CED. There is cerebral edema and a single focus of hemorrhage in the R cerebral hemisphere, adjacent to the medial boundary of the R lateral ventricle and another in the lateral hypothalamic area. (B) Lipoplatin™ (0.9 µg cisplatin). The rat was euthanized 4 days after CED. There is extensive necrosis with a dense infiltrate of foamy macrophages. (C) Lipoplatin™ (0.9 µg cisplatin) euthanized on d 4. There is a 6×4 mm zone of advanced necrosis with a peripheral zone of microglial reaction. There is edema of the neurophil and vacuolization, but no inflammatory response. (D) and (E) Lipoplatin™ “hollow” liposomes (100×). There is a 7×4 mm zone of hemorrhage associated with necrosis and edema of the neuropil. (E) There is an infiltrate of macrophages and hyperplasia of endothelial cells of adjacent small vessels. (F) Free cisplatin (3 µg), rats euthanized on d 7. Higher power (400×) view of (Fig. 4H). There is hemorrhage, necrosis and infiltration with lipid laden macrophages. Scattered larger blood vessels show early fibrin deposition in the walls.

**Figure 4:**
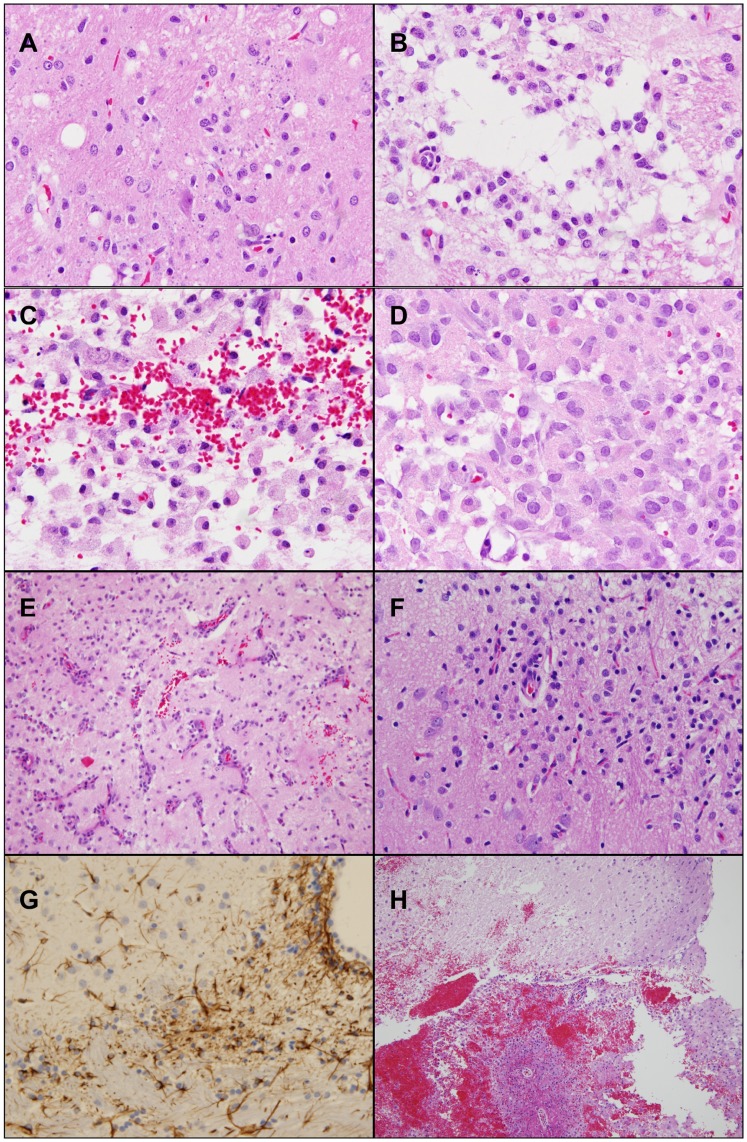
Neuropathologic changes associated with i.c. CED of cisplatin containing CHEMS liposomes (A-E), their “hollow” counterparts (F), or free cisplatin (H). (H&E) stained coronal sections at 400× magnification unless otherwise noted. (A) and (B) CHEMS – cisplatin, (4.8 µg), euthanized at 2 wks. (A) Although no necrosis is seen there are clear, possibly lipid containing vacuoles scattered reactive astrocytes and a mild infiltrate of macrophages. (B) There is focus of incipient necrosis and a moderate infiltrate of macrophages. Neurons consistent with late neuronal injury, proliferation of astrocytes and ependymal cells along the wall of the ventricle are also seen, but not in this photomicrograph. (C) and (D) CHEMS – cisplatin (9 µg), euthanized on d 10. There is focal intense inflammation with large numbers of macrophages, scattered lymphocytes, hemorrhage and necrosis and (E) prominent neovascularization. (F) and (G) “Hollow” CHEMS liposomes, euthanized on d 10. There is prominent necrosis with an intense inflammatory response consisting of lymphocytes and macrophages. Small vessels are engorged with blood and show reactive endothelial cells. (G) GFAP immunostaining revealed paraventricular foci of reactive astrogliosis. (H) Free cisplatin (3 µg), euthanized on d 7. Low power view (100×) shows a prominent focus (7×7 mm) of confluent hemorrhage with disruption of adjacent necrotic white matter.

It is noteworthy that Lipoplatin™ and its “hollow” counterpart showed equivalent *in vitro* cytotoxicity against F98 glioma cells (S.F. 0.51 *versus* 0.43) following a 4 hr incubation. This was attributed to the intrinsic toxicity of the “hollow” liposomes and the slow release of cisplatin from Lipoplatin™. The *in vitro* cytotoxicity of cisplatin containing CHEMS liposomes was 10× greater than the “hollow” counterpart (S.F. 0.05 versus 0.59). Free cisplatin at a concentration of 3 µg/ml showed the most potent *in vitro* cytotoxicity (S.F. 0.0002) and this correlated with the marked *in vivo* neurotoxicity of the free drug (Fig. 3H and 4F).

### Neurotoxicologic Studies

Unexpectedly, rats that received the highest doses of Lipoplatin™ (1.8 and 2.7 µg cisplatin) by CED showed acute neurotoxicity, which was manifested as soon as the effects of anesthesia had worn off. These animals clearly were in acute distress, as evidenced by vocalization suggesting that they were in pain, hyperactivity and sensitivity to touch. They were re-anesthetized in the hope that when they again came out of anesthesia, their clinical status would have improved. Since this was not the case, they then were euthanized. The brains of these animals showed one or more foci of acute hemorrhage (Fig. 3A) at sites other than along the needle track. Rats that received Lipoplatin™, containing 0.9 or 0.45 µg of cisplatin, which were euthanized 4 days following administration, showed a spectrum of changes ranging from mild to severe with hemorrhage, extensive necrosis, with or without infiltrates of foamy macrophages (Fig.3B and C). Rats that received the “hollow” counterparts of Lipoplatin™ clinically did not appear to be sick. However, the brains of these animals, euthanized d 4 following administration, showed hemorrhage with associated necrosis, edema of the neuropil and an infiltrate of macrophages (Fig. 3D and E). In summary, Lipoplatin™ and its “hollow” counterpart proved to be highly neurotoxic when administered i.c. by CED at these doses. This in part was due to the intrinsic toxicity of the liposomes themselves, together with the neurotoxic effects of cisplatin itself.

In contrast, the brains of half of the rats that received CHEMS liposomes at doses of 4.8, 7.2 or 9.6 µg of cisplatin showed minimal histopathologic abnormalities at 2 wks following administration. In some animals there were focal accumulations of clear vacuoles that presumptively may have contained lipid (Fig. 4A), and in others there were scattered reactive astrocytes and astrocytosis with necrosis and incipient necrosis, infiltrates of macrophages (Fig. 4B), and evolving reactive eosinophilic neurons, which were consistent with late neuronal injury. In a second study with CHEMS liposomes, containing 3, 6 or 9 µg of cisplatin rats were euthanized at d 10 following administration by CED. Again, there was a spectrum of changes ranging from normal in the brains of some rats, and focal areas of hemorrhage in others that received the lowest dose, to focal accumulations of lymphocytes and macrophages (6 µg of cisplatin) to focal areas of intense inflammation (Fig. 4C and D) with large numbers of lymphocytes and macrophages, hemorrhage (Fig. 4C) and prominent neovascularization (Fig. 4E) and necrosis (9 µg). Based on these findings, it was concluded that the 3, 4.8 or 6 µg doses of cisplatin of the CHEMS liposomes appeared to be reasonably well tolerated following i.c. CED although there were a variety of neuropathologic findings ranging from none to moderate. On the other hand, the 9 µg dose produced severe neuropathologic changes.

The brains of rats that received “hollow” CHEMS liposomes, which were euthanized at d 10 following administration, showed prominent necrosis with an intense inflammatory response consisting of lymphocytes and macrophages (Fig. 4F). Immunostaining for glial fibrillary acidic protein (GFAP) revealed foci of reactive astrogliosis in the paraventricular area (Fig. 4G). The brains of rats that received either 6 or 3 µg of free cisplatin, which were euthanized on d 7, showed the most severe changes of any that were seen in the present study. These included prominent confluent areas of hemorrhage with white matter necrosis (Fig. 4H), infiltrates of macrophages and early fibrin deposition in the walls of blood vessels (Fig. 3F).

### Biodistribution and Tumor Retention of Liposomal Cisplatin in F98 Glioma Bearing Rats

The amounts of platinum retained in the brain tumors of rats were 46.6±4.2% at 1 h following CED of CHEMS liposomes and 26.6±1.2% at 24 h. The tumor concentration was 4.4 µg/g immediately following CED of CHEMS liposomes containing 9.6 µg of cisplatin and 2.8 µg/g at 24 h (P = 0.05, Table 3). This was significantly higher (P = 0.03) than the drug concentrations in normal brain (0.1 to 0.8 µg/g). The tumor Pt concentration was comparable to that detected immediately following CED of carboplatin when adjusting for the higher dose of the latter [5]. Furthermore, based on these studies [5], the concentrations in extracranial tissues would have been in the undetectable range (<0.01 µg/g).

**Table 3:** Biodistribution of liposomes following CED to F98 glioma bearing rats.

	Mean cisplatin concentrations (µg/g) ± SD^c^				
Group^a^	Tumor	Brain around^b^ tumor (BAT)	Brain (R)	Brain (L)	Blood^d^
0 h	4.4±0.9	2.2±1.4	0.3±0.4	0.6±0.7	<0.01
24 h	2.8±0.2^c^	1.3±1.0	0.1±0.2	0.8±0.3	<0.01

a F98 glioma cells were implanted into the right caudate nucleus of 8 Fischer rats. Twelve to 14 d later, they received CED of cisplatin containing CHEMS liposomes (9.6 µg in 10 µl over 30 min) and were euthanized either immediately following (t = 0 h.) CED or 24 h later. The tumors, BAT and normal brain and blood samples were collected and Pt concentrations were determined by ICP-OES.

b Brain around tumor arbitrarily included an area of 1 mm beyond the dissected margins of the tumor.

c Means and standard deviations (SD) were calculated on values obtained from 4 rats for each time point.

d The P-value<0.05 compared to that determined at t = 0. Other than this, there were no significant differences in the 0 and 24 h Pt values.

## Discussion

The purpose of the present study was to evaluate two liposomal formulations of cisplatin as potential candidates for future *in vivo* therapeutic studies using the F98 rat glioma model. The first, Lipoplatin™, was found to be highly neurotoxic following i.c. CED to non-tumor bearing Fischer rats. Therefore, we designed, prepared and characterized a novel liposomal carrier with the cisplatin bound to CHEMS as a ligand in the outer lipid layer. This formulation addressed two common problems associated with liposomal drug delivery, the efficiency of encapsulation and drug release [26]. Failure of the liposomes to release the encapsulated drug would compromise its therapeutic efficacy. To obviate this problem, cisplatin was added to the CHEMS liposomes *after* their preparation in order to allow for the cisplatin-CHEMS complex to form on the outer lipid layer. The residual negative ζ potential of the nanoparticles possibly was due to the negative charge of the mPEG-DSPE conjugate.

The acute neurotoxicity of Lipoplatin™ and the “hollow” counterpart was completely unexpected since Lipoplatin™ has been shown to be safe and therapeutically effective following i.v. infusion in patients with NSCLC [8], [9]. Extensive *in vitro* [25] and *in vivo* [27] studies on Lipoplatin™ have been carried out by Paquette and his research team using the F98 glioma model. Lipoplatin™ showed only a slight reduction in *in vitro* tumoricidal activity compared to low concentrations of free cisplatin, and that this was enhanced when it was combined with X-irradiation [25]. Based on these promising results, *in vivo* studies were initiated with Lipoplatin™ and Lipoxal™, which contained oxaliplatin [27]. These were administered to F98 glioma bearing rats by intracarotid infusion either alone or in combination with a single 15 Gy dose of X-irradiation. Animals that received Lipoplatin™ showed a marked reduction in toxicity compared to cisplatin, and the MST was 30.2 d, compared to 22.0 d for untreated controls and 13.3 d for cisplatin treated rats whose deaths were attributable to drug toxicity. However, as reported by Elleaume and her research team [28], [29], free cisplatin administered by i.c. CED (6 µg in 20 µL) to F98 glioma bearing rats, resulted in a MST of 59 d with a 13% cure rate with no clinical evidence of neurotoxicity compared to a MST of 24 d for untreated controls. However, neuropathologic studies to determine the toxicity of cisplatin were not carried out and based on our own observations; we would expect that this would have produced neurotoxic effects. Even better results have been obtained by i.c. CED of carboplatin in combination with 6 MV photons [5], [30]–[32] without any histopathologic evidence of neurotoxicity [5]. As we have shown in the present study, free cisplatin at doses of either 3 or 6 µg produced severe neuropathologic changes consisting primarily of hemorrhage and necrosis at the site of administration. This is in striking contrast to the minimal neuropathologic changes observed, following i.c. CED of 20 µg carboplatin or infusion by means of Alzet osmotic pumps (84 µg) [5].

Cisplatin is subject to import mechanisms through the cell membrane using copper transporters limiting its cytoplasmic uptake [7], whereas its formulation into Lipoplatin™ appears to yield nanoparticles that greatly enhance its cytoplasmic uptake through fusion with the cell membrane or by endocytosis [10]. The fusogenic properties of Lipoplatin™, along with its tumor concentration after i.v. administration to patients, resulted in a 40-fold higher concentration in tumors compared to adjacent normal tissues in surgically resected specimens obtained from patients with NSCLC [6]. This resulted in a significant increase in its therapeutic efficacy [9]. However, as shown in the present study, its fusogenic properties may make it unsuitable for direct i.c. CED to the brain. The acute neurotoxicity observed almost immediately following the administration of Lipoplatin™ and its hollow liposome counterparts, which consisted of pain and hypersensitivity to touch, may have been due to cerebral edema and hemorrhage. Intravenous administration of Lipoplatin™ to treat brain tumors is currently under investigation and animal studies suggest that it crosses the BBB to a greater extent than free cisplatin (Boulikas, unpublished data).

There is very little in the neuropathology [33], neurotoxicology [34], [35] and nanotoxicology literature [36], [37] relating to the neurotoxic effects of drug containing liposomes administered i.c. by CED. Liposomal size and shape are critical determinants as to whether they can traverse the BBB [38]. Direct i.c. CED completely bypasses the BBB, and therefore, particle size may have been less important. However, surface composition and physical properties such as charge and ζ potential and their drug payload can be of the critical importance in determining whether they will have a direct neurotoxic effect and the former may explain the neurotoxicity of the “hollow” liposomes. The minimal neuropathologic changes seen within the brains of rats dying immediately following i.c. by CED of Lipoplatin™, and the severity of their acute neurologic symptoms suggest that acute cerebral edema was responsible. The changes seen in the brains of animals that were euthanized at 4 d following administration suggest that these were due to direct toxic effects on gray and white matter, which possibly may have been due to oxidative stress [10]. Turning to the neuropathologic changes seen following i.c. CED of CHEMS liposomes and their “hollow” counterparts liposomes in the present study, these ranged from none to moderate to severe for both the lower and higher dose. These may have been due to either the rapid or slow release of cisplatin in the case of the former, combined with the intrinsic toxicity of the CHEMS “hollow” liposomes. These findings precluded carrying out any therapy studies in F98 glioma bearing rats.

Bankiewicz and Dickinson [12]–[16], [20] and their respective research teams, have had the most experience of any investigators who have administered drug containing liposomes i.c. by CED. Most recently, they have evaluated CPT-11 (Irinotecan) containing liposomes in canines with spontaneous intracranial tumors. There was clear evidence of therapeutic efficacy, as determined by imaging studies that revealed a reduction in tumor volume, the presence of tumor necrosis and a change in tumor morphology consistent with a drug effect without any evidence of toxicity. Kawabata and his research team recently have evaluated sodium borocaptate, containing transferrin targeting liposomes, which were administered i.c. by CED, as potential boron delivery agents for neutron capture therapy of F98 glioma bearing rats [39]. These liposomes, which also contained an iodine contrast agent, were well tolerated clinically and imaging studies revealed precise localization at the site of the tumor with delivery of a high boron payload. However, no neuropathologic studies were carried out to determine if they were neurotoxic. Similarly, Brenner and his research team recently have evaluated rhenium-186 containing liposomes, which also were administered i.c. by CED to U87 glioma bearing rats [40]. The endpoint of these studies was prolongation of median survival time, which was significantly increased compared to that of rats that received non-radioactive liposomes with minimal neurotoxicity.

All of these studies suggest that i.c. CED of liposomes may have therapeutic efficacy. However, we do not believe that therapy studies with Lipoplatin™ or cisplatin containing CHEMS liposomes are warranted at this time, especially since such good survival data have been obtained in the F98 glioma model using CED or Alzet pump delivery of free carboplatin [5], [27], [28]. Our findings suggest that further refinements in the design and formulation of cisplatin containing liposomes will be required before they can be administered i.c. by CED for the treatment of brain tumors and that a formulation, such as Lipoplatin™, which may be safe when given systemically, may be highly neurotoxic when administered directly into the brain. Liposomal formulations of other cytoreductive therapeutic agents for the treatment of cancer [41], and more specifically of brain tumors, may have certain advantages [20]. However, at this point in time no such agents have been used clinically. Further studies using them are being pursued and it remains to be determined if indeed they will have clinical utility.
